# Abscission in plants: from mechanism to applications

**DOI:** 10.1007/s44307-024-00033-9

**Published:** 2024-08-09

**Authors:** Jiahuizi Li, Shihao Su

**Affiliations:** School of Agriculture and Biotechnology, Sun Yat-sen University, Shenzhen, 518107 China

**Keywords:** Plant abscission, Abscission zone, Molecular mechanism, Agriculture

## Abstract

Abscission refers to the natural separation of plant structures from their parent plants, regulated by external environmental signals or internal factors such as stress and aging. It is an advantageous process as it enables plants to shed unwanted organs, thereby regulating nutrient allocation and ensuring the dispersal of fruits and seeds from the parent. However, in agriculture and horticulture, abscission can severely reduce crop quality and yield. In this review, we summarize the recent advances in plant abscission from the perspectives of developmental and molecular biology, emphasizing the diverse regulatory networks across different plant lineages, from model plants to crops. The sophisticated process of plant abscission involves several overlapping steps, including the differentiation of the abscission zone, activation of abscission, tissue detachment, and formation of a protective layer. Finally, we discuss the potential applications of physiological modifications and genetic manipulations of plant abscission in sustainable agriculture in the future.

## Introduction

The word “abscission” refers to “removal or cutting away”, derived from the Latin “*abscissionem*”. In botany, it refers to the separation of plant structures, such as leaves, branches, flowers, or fruits, away from the parent plant owing to environmental changes (Estornell et al. 2013). These changes can be induced by a series of developmental (i.e., aging or maturation) or external environmental signals, including abiotic (drought, dark, hypoxia, extreme temperature, and nutrition limitation) and biotic stresses (mainly diseases or pests) (Reichardt et al. 2020; Li et al. 2021a; Goto et al. 2022; Meng et al. 2023; Ruiz et al. 2001; Patharkar et al. 2017).

From the perspectives of ecology and evolution, abscission is a beneficial process as it helps the parent plant discard unwanted parts such as wilted flowers or leaves, and hence regulates nutrient allocation. For example, leaf abscission can be triggered by drought, which may enable the plant to prepare well for subsequent occurrences of drought by reducing the leaf area for transpiration. Mobile nutrients, including those belonging to the three main nutrients classes (nitrogen, phosphorus, and potassium), are drawn out of the unhealthy old leaves before abscission to facilitate the continued growth of healthy young tissues (Patharkar and Walker 2016). In addition, leaf abscission can be triggered by pathogens in *Arabidopsis thaliana*, enabling the plants to shed infected leaves and eliminate the spread of the disease to healthy tissues (Patharkar et al. 2017). In forest and savanna ecosystems, abscised leaf litter plays key roles in nutrient and carbon cycling and forms a protective layer on the soil surface, thereby regulating the soil microclimate (Villalobos-Vega et al. 2011; Zhou et al. 2019). Abscission is also a key strategy for plant reproductive success, as it ensures the separation of fruits, which further crack to disperse the seeds. The seed abscission process largely relies on the wind, an important dispersal vector (Ferrándiz 2002; Schippers and Jongejans 2005).

However, in agriculture and horticulture, abnormal abscission is closely associated with severe reduction in crop quality and yield. In wild species of domesticated crops, seed shattering is an essential characteristic to ensure the survival of the next generation, whereas it causes major yield loss in crops harvested by humans. Therefore, our ancient farmers domesticated these wild species by collecting seeds from plants with favorable traits, including the loss of shattering, and produced non-shattering cultivated crops (Alam and Purugganan 2024). In addition, many fruit trees such as apple, pear, litchi, and *citrus*, suffer from flower and fruit abscission, which functions as a double-edged sword: excessive abscission leads to yield loss, while rational control of shedding may increase yield and improve fruit quality (Kon et al. 2023; Webster 2002; Zhao and Li 2020; Dutta et al. 2023).

**Fig. 1:**
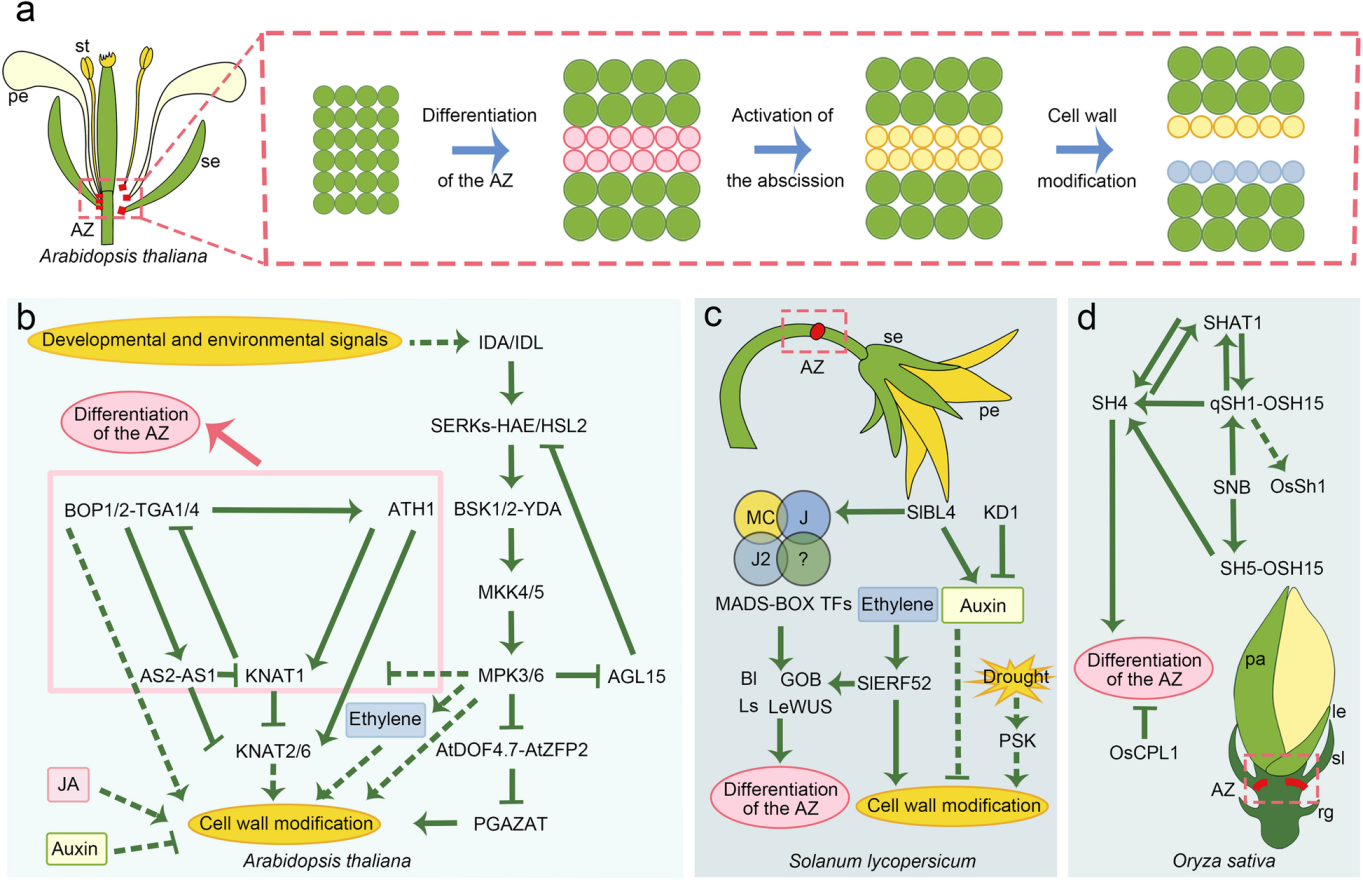
The process and molecular control of plant abscission. **a** Three overlapped steps during floral organ abscission in Arabidopsis. Abscission zone: AZ; se: sepal; pe: petal; st: stamen; green circles: other cells; red circles: differentiated cells in AZ; yellow circles: activated cells in AZ; blue circles: transdifferentiated cells on the surface of receptacle. (b-d) Molecular control of plant abscission in *Arabidopsis thaliana* (**b**), *Solanum lycopersicum* (**c**) and *Oryza sativa* (**d**). Dashed green lines: hypothetical pathways

Here, we review the recent advances in plant abscission from the perspectives of developmental and molecular biology, with an emphasis on the diverse regulatory networks in different plant lineages, from model plants to crops (Fig. 1; Table 1). The abscission process consists of several overlapping steps, including differentiation of the abscission zone, activation of abscission, tissue detachment, and formation of a protective layer (Fig. 1a). In the final part, we discuss the potential applications of physiological modifications and genetic manipulations of plant abscission for crop breeding. This review aims to enrich our understanding of the molecular regulatory networks involved in plant abscission and provide guidance for sustainable agriculture in the future.

**Table 1:** Summary of key regulators of plant abscission in *Arabidopsis thaliana*, *Solanum lycopersicum* and *Oryza spp*

**Species**	**Gene**	**Gene family**	**Functions in plant abscission**	**Reference**
***Arabidopsis thaliana***	*ATH1*	BELL	Promote the development of AZ; Promote the separation step of organ abscission	Gomez-Mena and Sablowski 2008
	*AS1*	MYB	Regulates the position of AZ	Gubert et al. 2014b
	*AS2*	LOB	Regulates the position of AZ	Jun et al. 2010
	*BOP1*	NPR1	Promote the development of AZ	McKim et al. 2008
	*BOP2*	NPR1	Promote the development of AZ	McKim et al. 2008
	*KNAT1/BP*	KNOX	Promote the development of AZ; Inhibit the separation step of organ abscission	Shi et al. 2011; Butenko et al. 2012
	*AGL42/FYF*	MADS-box	Inhibit the development of AZ; Inhibit the separation step of organ abscission	Chen et al. 2011
	*TGA1*	bZIP	Promote the development of AZ	Wang et al. 2019b
	*TGA4*	bZIP	Promote the development of AZ	Wang et al. 2019b
	*PNY*	BELL	Inhibit the development of AZ	Andrés et al. 2015
	*OFP1*	OFP	Promote the development of AZ	Zhang et al. 2018
	*STM*	KNOX	Promote the development of sepal AZ	Song et al. 2020
	*AGL15*	MADS-box	Inhibit the separation step of organ abscission	Patharkar and Walker 2015
	*AGL71/FYL1*	MADS-box	Inhibit the separation step of organ abscission	Chen et al. 2022
	*AtDOF2.3/CDF4*	DOF	Promote the separation step of organ abscission	Xu et al. 2020
	*AtDOF4.7*	DOF	Inhibit the degradation of the abscission layer	Wang et al. 2016
	*AtZFP2*	ZFP	Inhibit the degradation of the abscission layer	Wei et al. 2010
	*BIR1*	RLK	Inhibit the separation step of organ abscission	Taylor et al. 2019
	*CST*	RLCK	Inhibit the separation step of organ abscission	Burr et al. 2011
	*EVR/SOBIR1*	LRR-RLK	Inhibit the separation step of organ abscission	Leslie et al. 2010; Gubert et al. 2014a
	*HAE*	LRR-RLK	Promote the separation step of organ abscission	Jinn et al. 2000
	*HSL2*	LRR-RLK	Promote the separation step of organ abscission	Cho et al. 2008
	*IDA*	IDL	Promote the separation step of organ abscission	Cho et al. 2008
	*IDL*	IDL	Promote the separation step of organ abscission	Stenvik et al. 2008
	*KNAT2*	KNOX	Promote the separation step of organ abscission	Ragni et al. 2008
	*KNAT6*	KNOX	Promote the separation step of organ abscission	Belles-Boix et al. 2006
	*BSK1*	BR-signaling kinase	Promote the separation step of organ abscission	Galindo-Trigo et al. 2024a
	*BSK2*	BR-signaling kinase	Promote the separation step of organ abscission	Galindo-Trigo et al. 2024a
	*YDA/MAPKKK4*	MAPKKK	Promote the separation step of organ abscission	Galindo-Trigo et al. 2024a
	*MKK4*	MAPKK	Promote the separation step of organ abscission	Cho et al. 2008
	*MKK5*	MAPKK	Promote the separation step of organ abscission	Cho et al. 2008
	*MPK3*	MAPK	Promote the separation step of organ abscission	Cho et al. 2008
	*MPK6*	MAPK	Promote the separation step of organ abscission	Cho et al. 2008
	*NEV*	ARF GAPs	Promote the separation step of organ abscission	Gubert et al. 2014a
	*SERK1*	LRR-RLK	Promote the separation step of organ abscission	Meng et al. 2016
	*SERK2*	LRR-RLK	Promote the separation step of organ abscission	Meng et al. 2016
	*SERK3*	LRR-RLK	Promote the separation step of organ abscission	Meng et al. 2016
	*SERK4*	LRR-RLK	Promote the separation step of organ abscission	Meng et al. 2016
	*WRKY57*	WRKY	Promote the separation step of organ abscission	Galindo-Trigo et al. 2024b
	*ADPG1*	PG	Promote the degradation of the abscission layer cell wall	Ogawa et al. 2009
	*ADPG2/PGAZAT*	PG	Promote the degradation of the abscission layer cell wall	Ogawa et al. 2009
	*QRT2*	PG	Promote the degradation of the abscission layer cell wall	Ogawa et al. 2009
***Solanum lycopersicum***	*Bl*	MYB	Promote the development of AZ	Nakano et al. 2013
	*GOB*	NAC	Promote the development of AZ	Nakano et al. 2013
	*J-2*	MADS-box	Promote the development of AZ	Roldan et al. 2017
	*LeWUS*	WOX	Inhibit the development of AZ	Nakano et al. 2013
	*Ls*	GRAS	Inhibit the development of AZ	Nakano et al. 2013
	*MC*	MADS-box	Promote the development of AZ	Nakano et al. 2012
	*SlBL4*	BELL	Promote the development of AZ	Yan et al.^2021^
	*SlERF52*	AP2/ERF	Promote the development of AZ;Promote the separation step of abscission	Nakano et al. 2014
	*SlIDA*	IDL	Promote the separation step of abscission	Lu et al. 2023
	*SlIDL2*	IDL	Promote the separation step of abscission	Lu et al. 2023
	*SlIDL3*	IDL	Promote the separation step of abscission	Lu et al. 2023
	*SlIDL4*	IDL	Promote the separation step of abscission	Lu et al. 2023
	*SlIDL5*	IDL	Promote the separation step of abscission	Lu et al. 2023
	*SlHSL6*	LRR-RLK	Promote the separation step of abscission	Lu et al. 2023
	*SlHSL7*	LRR-RLK	Promote the separation step of abscission	Lu et al. 2023
	*SlKD1*	KNOX	Promote the separation step of abscission	Lu et al. 2023
	*SlPhyt2*	phytaspase	Promote the separation step of abscission	Reichardt et al. 2020
	*PSK*	Phytosulfokines	Promote the separation step of abscission	Reichardt et al. 2020
	*SlARF10A*	ARF	Inhibit the separation step of abscission	Damodharan et al. 2016
	*SlBEL11*	BELL	Inhibit the separation step of abscission	Dong et al. 2024
	*SlHXK1*	HXK	Inhibit the separation step of abscission	Li et al. 2020
	*SlHB15A*	HD-Zip	Inhibit the separation step of abscission	Liu et al. 2022
	*SlPIN1*	PIN	Inhibit the separation step of abscission	Shi et al. 2017
	*SlFYFL*	MADS-box	Inhibit the separation step of abscission	Xie et al. 2014
	*SlCEL1*	CEL	Promote the degradation of the abscission layer cell wall	Campillo and Bennett 1996
	*SlCEL2*	CEL	Promote the degradation of the abscission layer cell wall	Campillo and Bennett 1996
	*SlCEL3*	CEL	Promote the degradation of the abscission layer cell wall	Campillo and Bennett 1996
	*SlCEL4*	CEL	Promote the degradation of the abscission layer cell wall	Campillo and Bennett 1996
	*SlCEL5*	CEL	Promote the degradation of the abscission layer cell wall	Campillo and Bennett 1996
	*SlCEL6*	CEL	Promote the degradation of the abscission layer cell wall	Campillo and Bennett 1996
	*TAPG1*	PG	Promote the degradation of the abscission layer cell wall	Kalaitzis et al. 1997
	*TAPG2*	PG	Promote the degradation of the abscission layer cell wall	Kalaitzis et al. 1997
	*TAPG4*	PG	Promote the degradation of the abscission layer cell wall	Kalaitzis et al. 1997
	*TAPG5*	PG	Promote the degradation of the abscission layer cell wall	Kalaitzis et al. 1997
***Oryza spp.***	*GL4*	MYB	Promote the development of AZ	Wu et al. 2023b
	*ObSH3*	YABBY	Promote the development of AZ	Lv et al. 2018
	*OsSh1*	YABBY	Promote the development of AZ	Lin et al. 2012
	*qCSS3*	-	Promote the development of AZ	Tsujimura et al.^2019^
	*qSH1*	BELL	Promote the development of AZ	Konishi et al. 2006
	*qSH3*	-	Promote the development of AZ	Inoue et al. 2015
	*SH4/SHA1*	trihelix	Promote the development of AZ	Li et al. 2006
	*SH5*	BELL	Promote AZ development; Inhibiting lignin biosynthesis	Yoon et al. 2014
	*SHAT1*	AP2/ERF	Promote the development of AZ	Zhou et al. 2012
	*OsSNB/SSH1*	AP2/ERF	Promote the development of AZ and vascular bundle	Jiang et al. 2019
	*OsCPL1*	CTD phosphatase-like gene	Inhibit the differentiation of abscission layer	Ji et al. 2010
	*OsGRF4*	armadillo/beta-catenin repeat	Inhibit the differentiation of abscission layer	Sun et al. 2016
	*4CL3*	CoA ligase	Promote lignin deposition in the AZ	Wu et al. 2023a
	*OsCAD2/GH2*	CAD	Promote lignin deposition in the AZ	Ning et al. 2023; Yoon et al. 2017
	*OgSH11*	MYB	Inhibiting lignin biosynthesis	Ning et al. 2023
	*OsCel9D*	CEL	Promote the degradation of;the abscission layer cell wall	Nunes et al. 2014
	*OSH15*	KNOX	Inhibiting lignin biosynthesis	Yoon et al.^2017^
	*OsXTH8*	XTH	Promote the degradation of the abscission layer cell wall	Nunes et al. 2014
	*SHA1*	Trihelix	Promote the degradation of the abscission layer cell wall	Lin et al. 2007
	*ZlqSH1a*	BELL	Promote the development of AZ	Xie et al. 2022
	*ZlqSH1b*	BELL	Promote the development of AZ	Xie et al. 2022

### Where to drop: the abscission zone

The location where abscission occurs, known as the abscission zone (AZ), is determined in the early developmental stages. AZs are present in various plant structures, including petioles, pedicels, and floral organs. Cell morphology in the AZ typically exhibits characteristics such as a smaller size compared to neighboring non-abscessed cells, denser protoplasm, increased cell density, and more complex plasmodesmata (Sexton and Roberts 1982). The number of cell layers in the AZ varies significantly across tissues and species. The floral organs of* A. thaliana* have 4-6 layers of cells in their AZ (McKim et al. 2008), tomato pedicels have 5-10 layers (Roberts et al. 1984), and the AZ at the leafstalks of *Sambucus nigra* consists of 50 layers of cells (Taylor and Whitelaw 2001). However, actual cell separation does not occur uniformly across the AZ but is typically limited to several distal cell layers, referred to as the abscission layer (Roberts et al. 1984). The abscission process has been described as a multistage process: (1) differentiation of the AZ, (2) activation of the abscission process in response to developmental and environmental signals, and (3) cell wall modification, followed by cell detachment and the formation of a rigid protective layer (Patterson 2001; Estornell et al. 2013).

### Differentiation of the AZ

Many genes involved in the formation of AZ within floral organs, such as petals, sepals, and stamens, have been identified in *Arabidopsis* (Fig. 1a, b). *BLADE-ON-PETIOLE 1/2* (*BOP1/2*) are *NONEXPRESSOR OF PATHOGENESIS RELATED GENES 1* (*NPR1*)*-like* genes that redundantly regulate plants developmental patterning and facilitate the formation of AZ (Hepworth et al. 2005; Su et al. 2023). The *bop1 bop2* double mutant fails to develop the anatomical structures of the AZ at the floral organ boundaries, leading to defects in organ abscission (McKim et al. 2008). In tobacco, a homolog of *BOP*, *NtBOP2*, regulates corolla abscission by inhibiting the longitudinal elongation of cells in the corolla AZ (Wu et al. 2012). Similarly, in tomatoes, CRISPR mutants of three *BOP* genes result in the failure of petal abscission (Xu et al. 2016). In legume species, *Medicago truncatula*, *Pisum sativum* and *Lotus japonicus*, *BOP* orthologs are necessary for the abscission of vegetative and reproductive structures (Couzigou et al. 2016). These findings support the important, conserved function of *BOP* in promoting AZ differentiation in eudicots.

*Arabidopsis* BOP1 and BOP2 form homodimers or heterodimers that enable them to activate the transcription of *ASYMMETRIC LEAVES 2* (*AS2*) during leaf development (Jun et al. 2010). *AS2* encodes an LBD transcription factor that acts in conjunction with the MYB transcription factor AS1. AS1 is also involved in the proper placement of the floral organ AZs and, together with AS2, forms a transcriptional complex that specifically binds to the CWGTTD motifs in the promoters of *KNOTTED1-LIKE HOMEODOMAIN* (*KNOX*) genes, such as *KNOTTED-LIKE FROM ARABIDOPSIS THALIANA 1/2/6* (*KNAT1/2/6*, *KNAT1* is also known as *BREVIPEDICELLUS* (*BP*)), resulting in the repression of their expression (Guo et al. 2008). KNAT1 inhibits floral organ abscission by limiting AZ cell size and number (Shi et al. 2011). In tomatoes, the *KNOX* gene *KD1* is involved in the regulation of tomato flower pedicel abscission via the modulation of auxin concentration and response in the AZ (Ma et al. 2015). In *Litchi chinensis*, a tropical fruit originating from south China, *LcKNAT1* is expressed in the fruitlet AZ, and ectopic expression of *LcKNAT1* in tomatoes leads to delayed pedicel abscission (Zhao et al. 2020). These results reveal a shared role of KNOX proteins as negative abscission regulators. In addition, BOP1/2 can form complexes with the transcription factors TGACG-BINDING FACTOR 1/4 (TGA1/4), leading to the direct activation of the *BEL1-LIKE* (*BELL*) gene, *ARABIDOPSIS THALIANA HOMEOBOX GENE1* (*ATH1*) (Khan et al. 2015). Both BELL and KNOX proteins belong to the three-amino-acid loop extension (TALE) protein family and share similar structures and functions in diverse developmental processes. ATH1 positively regulates stamen abscission and, together with its partners KNAT2/6, contribute to the differentiation of floral organ AZs (Crick et al. 2022). Additionally, TALE homeodomain transcription factors (ATH1, KNAT2/6) and BOP1/2 work together during lignin deposition to promote the expression of hydrolytic enzymes involved in cell separation (Crick et al. 2022). BOP1/2 contribute to cell separation via activation of *ATH1* and *KNAT2/6* or independently through the promotion of genes involved in cell separation (Crick et al. 2022).

Abscission occurs not only within floral organs but also in whole flowers, fruits, and branches. The tomato has been used as a model to study the mechanisms of AZ formation within the pedicel, where aborted flowers or ripe fruits are shed (Fig. 1c). Formation of the pedicel AZ in tomato requires the presence of at least three MADS-box transcription factors: JOINTLESS (J), JOINTLESS-2 (J-2, also known as SlMBP21), and MACROCALYX (MC) (Nakano et al. 2012; Roldan et al. 2017). Loss-of-function mutations in any of these genes result in the failure of pedicel AZ development. As MADS-box transcription factors assemble into core tetrameric protein complexes in the floral quartet model, J, J-2, and MC may also form tetramers with other MADS-box proteins, thereby serving as transcriptional activators that promote the development of the pedicel AZ (Liu et al. 2014). J, J-2, and MC activate the expression of pre-abscission-related genes, such as *BLIND* (*Bl*), *GOBLET* (*GOB*), *Lateral suppressor* (*Ls*), and a *WUSCHEL* homologue in tomato (*LeWUS*) (Nakano et al. 2012; Roldan et al. 2017; Nakano et al. 2013). The transcription of *J* is activated by BEL1-LIKE HOMEODOMAIN 4 (SlBL4) in vitro, supporting SlBL4’s role in fruit pedicel organogenesis and abscission (Yan et al.^2021^). However, the orthologs of these three MADS proteins in *Arabidopsis* are not related to pedicel abscission, and it remains unclear whether the functions of MADS transcription factors in the regulation of pedicel AZ development are conserved in other species or if they are specific to tomato and its relatives.

Preharvest fruit shattering occurs in many wild relatives of Poaceous crops, causing reduced yield and seed quality. Therefore, natural mutants with non-shattering trait were often selected during crop domestication (Yu et al. 2024). Although shattering positions vary among different Poaceous crops, they are often related to structures such as floral bracts and stem segments (Yu et al. 2024). In rice, the AZ consists of a layer of non-lignified cells surrounded by thick lignified cells (Wu et al. 2023b), and numerous shattering factors have been identified, including eight major factors: SUPERNUMERARY BRACT (SNB), QTL OF SEED SHATTERING IN CHROMOSOME 1 (qSH1), GRAIN SHATTERING QUANTITATIVE TRAIT LOCUS ON CHROMOSOME 4 (SH4), SH5, SHATTERING ABORTION1 (SHAT1), ORYZA SATIVA CTD PHOSPHATASE-LIKE 1 (OsCPL1), ORYZA BARTHII SEED SHATTERING 3 (ObSH3), and OgSH11 (Fig. 1d). These factors form a complicated network that regulates the expression of key lignin biosynthesis genes: *GOLD HULL AND INTERNODE2* (*GH2*)/*CINNAMYL-ALCOHOL DEHYDROGENASE 2* (*CAD2*) and *4-COUMARATE: COENZYME A LIGASE 3* (*4CL3*) (Wu et al. 2023a; Wu et al. 2023b). Notably, differential lignification that occurs in rice AZ formation may not be essential for shattering in many other Poaceae crops, such as sorghum and wheat, suggesting divergent genetic control of cereal shattering.

### Activation of the abscission process

In *A. thaliana*, the IDA-HAE/HSL2 signaling pathway regulates the initiation of floral organ abscission (Fig. 1b). *HEASA* (*HAE*) and *HEASA-LIKE2* (*HSL2*) encode two closely related leucine-rich repeating receptor-like kinases (LRR-RLKs) that redundantly regulate the process. INFLORESCENCE DEFICIENT IN ABSCISSION (IDA) and IDA-LIKE (IDL) are small peptides that positively regulate floral organ abscission process (Jinn et al. 2000; Stenvik et al. 2008). The *ida* mutants, despite possessing the ability to form AZ, fail to abscise floral organs, whereas ectopic expression of *IDA* results in earlier abscission (Butenko et al. 2003). IDA functions as a ligand to activate the heterodimerization and transphosphorylation of HAE/HSL2, together with SOMATIC EMBRYOGENESIS RECEPTOR KINASE (SERK) family members that function as co-receptors (Meng et al. 2016). The IDA-HAE/HSL2 signaling module has been identified in different species, including tomato, tobacco, soybean, *citrus*, rose, and litchi, indicating its conservation across eudicots (Lu et al. 2023; Ventimilla et al. 2021; Tucker and Yang 2012; Estornell et al. 2015; Singh et al. 2023; Ma et al. 2024; Wang et al. 2019a; Ying et al. 2016). Future studies should focus on the crosstalk between the IDA-HAE/HSL2 signaling module and plant abscission hormones (i.e., ethylene/ET) to fully understand the ET-dependent and -independent abscission pathways (Meir et al. 2019).

The activated HAE/HSL2-SERK complex subsequently induces the downstream mitogen-activated protein kinase (MAPK) signaling cascade through brassinosteroid signaling kinases (BSKs) and the MAPKKK, YODA (YDA, also known as MAPKKK4) (Galindo-Trigo et al. 2024a). The activated MAPK cascade further phosphorylates the downstream transcription factors and ultimately enhances hydrolase activity, thereby facilitating floral organ abscission (Cho et al. 2008). The MADS-box transcription factor AGAMOUS-LIKE15 (AGL15) is a direct target of MAPK. AGL15 acts as a negative regulator of *HAE* before phosphorylation but becomes a positive regulator when phosphorylated, thereby establishing a feedback loop for the regulation of floral organ abscission (Patharkar and Walker 2015). Another DNA binding with one finger (DOF) transcription factor, AtDOF4.7, is also a direct target of the MAPK cascade, which enables AtDOF4.7 to directly repress the expression of the abscission-related polygalacturonase (PG) gene, *PGAZAT* (Wang et al. 2016). In addition, ZINC FINGER PROTEIN2 (AtZFP2), a zinc-finger protein, is specifically expressed in the floral AZ, and its overexpression leads to delayed abscission. AtDOF4.7, which forms a transcriptional complex with AtZFP2, enhances the repression of *PGAZAT* (Wei et al. 2010). Moreover, HAE/HSL2 and other RLKs must be transported through the intracellular membrane system, an ADP-ribosylation factor, The GTPase-activating protein NEVERSHED (NEV), located in the trans-Golgi network (TGN), is required. Mutations in *nev* disrupt the TGN structure of the cells within the AZ, leading to organ abscission failure (Liljegren et al. 2009).

### Cell detachment and protective layer formation

In response to an abscission signal, the cells in the AZ secrete enzymes, including cellulases (CELs), polygalacturonases (PGs), and xyloglucan endotransglucosylase/hydrolases (XTHs), that modify and hydrolyze the cell wall (Wu et al. 2024). These enzymes degrade cell walls between adjacent cell layers, ultimately resulting in cell detachment.

CEL is responsible for cellulose degradation. In *Arabidopsis*, AtCEL6 promotes silique dehiscence by facilitating cell separation in the abscission layer (He et al. 2018). Six cellulase genes (*SlCEL1-6*) have been identified in tomato, all of which are associated with ET-dependent floral abscission (Del Campillo and Bennett 1996). In litchi, the HD-Zip transcription factor LcHB2 acts as a positive regulator of fruit abscission by directly activating the cellulase genes *LcCEL2* and *LcCEL8* (Li et al. 2019).

PG catalyzes the degradation of pectin. Floral organ abscission in *Arabidopsis* depends on the presence of PGs, and mutations in PGAZAT, ARABIDOPSIS DEHISCENCE ZONE POLYGALACTURONASE1 (ADPG1) and QUARTET2 (QRT2) lead to delayed fruit dehiscence (Ogawa et al. 2009). Hence, both PGAZAT and QRT2 have been used as abscission markers (González-Carranza et al. 2007). Organ abscission is regulated by a combination of jasmonic acid (JA), ET, and abscission acid (ABA), which, in part, promotes the expression of *QRT2* (Ogawa et al. 2009). Tomato PGs, such as *TAPG1/2/4/5*, are specifically expressed during the ET-induced abscission of leaves and flowers. Their expression is promoted by the APETALA2/ethylene responsive factor (AP2/ERF) transcription factor SlERF52, which acts downstream of J and MC (Kalaitzis et al. 1997; Nakano et al. 2014). Under drought stress, the small signaling peptide hormone, phytosulfokine (PSK) can induce an elevated expression of TAPG4, thereby promoting the abscission of tomato flowers and fruits (Reichardt et al. 2020).

XTH disrupts the xyloglucan chains and remodels the cellulose-xyloglucan complex structure. Accumulation of XTH in the AZ has been observed during abscission in various species, such as *Arabidopsis*, tomato, rose, litchi, cherry, and soybean, indicating the potential importance of XTH in organ abscission (Lashbrook et al. 2008; Tsuchiya et al. 2015; Singh et al. 2013; Ma et al. 2021a; Qiu et al. 2021; Tucker et al. 2007). Studies have also demonstrated the ET-responsiveness of the transcription of XTHs during abscission. In litchi, two ETHYLENE INSENSITIVE 3-LIKE (EIL) homologs, LcEIL2 and LcEIL3, which function as core transcription factors that activate various ET responses, directly activate the XTH genes *LcXTH4/7/19*, and mediate fruit abscission (Ma et al. 2021b). In the petal AZ of rose, *RbXTH3/5/6/12* can be rapidly induced by ET within hours (Singh et al. 2013). Similarly, in citrus leaves, *CitXTH1-3* levels are upregulated in the AZ after ET treatment (Agustí et al. 2009).

Apart from cell detachment, a protective layer is also formed on the surface of the distal end of the abscission layer to protect the plant from pathogen entry and water loss. Single-cell transcriptomics demonstrates that the *Arabidopsis* floral organ AZ is composed of two neighboring cell types with distinct cellular activities: the secession cells (SEC) of the separated organs produce a honeycomb structure of lignin, that serves as a mechanical brace to localize cell wall breakdown and spatially restrict the detachment of cells; While the residuum cells (REC) of the receptacle undergo *a de novo* specification of epidermal cells by recruiting two wall-hydrolyzing proteins, QRT2 and XTH28, thereby leading to the formation of a protective cuticle (Lee et al. 2018; Kim et al. 2019). Although the biochemical reactions catalyzed by these enzymes are relatively well understood, their regulation and coordination during abscission requires further investigation.

### Manipulation of plant abscission in agriculture

The percentage of flower and fruitlet abscission is closely related to yield, therefore, the artificial regulation of plant abscission is an important way to ensure production. Hexanal, a natural compound produced in plants after injury, has been shown to be effective in preventing preharvest fruit drop of many fruits. For example, in apples, the application of hexanal can effectively reduce ET biosynthesis and perception in the AZ, preventing cell wall degradation, and consequently minimizing fruit drop (Sriskantharajah et al. 2021). However, in tea production, an ET-releasing molecule, ethephon, is used to stimulate flower abscission and decrease the overall number of flowers, thereby improving tea yield and quality (Zhang et al. 2022a).

Post-harvest storage and transportation of fruits and vegetables often involve issues of fruit or leaf abscission, which negatively affects their commercial value. Preharvest spraying of calcium nanoparticles on grapes results in an increased calcium pectinate content in the AZ, leading to delayed pectin degradation, suppressed ET synthesis, and inhibition of grape berry abscission (Zhu et al. 2024). Post-harvest application of nordihydroguaiaretic acid (NDGA) or chitosan can effectively inhibit the activity of cell wall-degrading enzymes, thereby delaying the cellular detachment of the AZ (Zhu et al. 2022; Wu et al. 2021). In cut flowers, petal abscission is profoundly promoted by ET, and the application of ET inhibitors such as silver thiosulfate (STS) and 1-methylcyclopropene (1-MCP) significantly extends the vase life of flowers. STS effectively reduces the activity of the enzymes involved in cell wall hydrolysis in the AZ, whereas 1-MCP inhibits ET perception by suppressing ET receptor genes and enhancing antioxidant activity (Zhang et al. 2022b; Naing et al. 2022).

Mechanical harvesting is the future of modern agriculture practices. To improve harvest productivity, it is necessary to cultivate varieties suitable for mechanical harvesting. During the processing of vegetables such as canned tomato and pepper, removing the pedicels and calyx depends on farm labor and time. Moreover, the presence of stems may cause mechanical damage to the fruits during transportation. In tomato, mutations in J, J-2, and MC disrupt AZ formation, allowing for mechanical harvesting without physical wounding during transportation (Ito and Nakano 2015). In *Capsicum annuum* cultivation, the Mexican landrace UCD-14 presents an easy-destemming trait, possibly due to the presence and activation of the pedicel/fruit AZ. Multiple quantitative trait locus (QTLs) known to control *Arabidopsis* abscission are found to co-segregate with the stem removal traits in UCD-14 (Hill et al. 2023). In *Brassica napus*, premature silique dehiscence results in devastating yield loss during mechanical harvesting (Li et al. 2021). Recently, modulating the expression of the hemicellulase gene *BnMAN7A07* or knock-out of two *IDA* homologs, *BnaIDA-A07* and *BnaIDA-C06*, suppressed silique dehiscence, improved yield, and facilitated mechanical harvesting in *Brassica napus* (Li et al. 2021b; Geng et al. 2022). Since rapeseed flower fields serve as significant rural tourist attractions, CRISPR-mediated *BnaIDA* gene editing extends the flowering period and enhances their resistance to *Sclerotinia sclerotiorum*, thereby boosting tourism income (Wu et al. 2022).

### Future perspectives

Plant hormones play a crucial role in regulating plant abscission and their crosstalk has long been a subject of research interest. A widely acknowledged hypothesis suggests that ET promotes abscission, whereas auxins inhibit this process. For example, *litchi AUXIN RESPONSE FACTOR 5* (*LcARF*5) and *LcEIL3* are upregulated by ET and downregulated by auxins, which promote fruit abscission through the expression of *LcIDL*1 and *LcHSL2* (Ma et al. 2024). Auxins plays various roles at different stages of abscission. A recent study indicated that JA induces autophagy to promote petal abscission (Furuta et al. 2024). In addition, gibberellins, ABA, and brassinosteroids have been reported to participate in the regulation of abscission (Marciniak et al. 2018; Wu et al. 2023a; Kućko et al. 2023; Ma et al. 2021a, b). However, the interactions between plant hormones remain unclear. Therefore, further research on the crosstalk among the hormones that participate during abscission is warranted.

### Concluding remarks

In recent decades, notable progress has been made in the study of abscission in model plants, which has paved the way for translational research in crops and other non-model plants. The utilization of single-cell sequencing or spatially enhanced-resolution omics-sequencing (Stereo-seq) will enable the study of plant abscission at single-cell resolutions (Baysoy et al. 2023). Such technologies will facilitate the elucidation of heterogeneity within different AZ cell populations and provide novel insights into the developmental process of AZ, thereby establishing a genetic toolkit for the genetic manipulation of plant abscission in future agriculture. In the future, understanding the developmental timing of plant abscission will enable precise human-regulation of the process of abscission. Combined with precise plant genome editing tools (Xiong et al. 2023), it will be possible to optimize harvestability traits and design novel crop cultivars suitable for mechanized harvesting in the future.

## Data Availability

No datasets were generated or analyzed during the current study.
